# Integrative taxonomy reveals hidden species within a common fungal parasite of ladybirds

**DOI:** 10.1038/s41598-018-34319-5

**Published:** 2018-10-29

**Authors:** Danny Haelewaters, André De Kesel, Donald H. Pfister

**Affiliations:** 1 Department of Organismic and Evolutionary Biology, Harvard University, 22 Divinity Avenue, Cambridge, Massachusetts 02138 USA; 2 Present Address: Faculty of Science, University of South Bohemia, Branišovská 31, 37005 České Budějovice, Czech Republic; 3 Botanic Garden Meise, Nieuwelaan 38, 1860 Meise, Belgium

## Abstract

Our understanding of fungal diversity is far from complete. Species descriptions generally focus on morphological features, but this approach may underestimate true diversity. Using the morphological species concept, *Hesperomyces virescens* (Ascomycota, Laboulbeniales) is a single species with global distribution and wide host range. Since its description 120 years ago, this fungal parasite has been reported from 30 species of ladybird hosts on all continents except Antarctica. These host usage patterns suggest that *H. virescens* could be made up of many different species, each adapted to individual host species. Using sequence data from three gene regions, we found evidence for distinct clades within *Hesperomyces virescens*, each clade corresponding to isolates from a single host species. We propose that these lineages represent separate species, driven by adaptation to different ladybird hosts. Our combined morphometric, molecular phylogenetic and ecological data provide support for a unified species concept and an integrative taxonomy approach.

## Introduction

*What is a species?* This is a perennial question in evolutionary biology. The answer is complex and has been intensely argued for decades. Different species concepts corresponding to multiple biological properties provide a means to recognize, delineate and describe species. These properties include differences in morphological traits, nucleotide divergence and monophyly, reproductive isolation, ecological niches or adaptive zones, mate recognition or mating systems, geographic range, exclusive coalescence of alleles, etc. However, biologists from various research fields have advocated different and sometimes incompatible species concepts, leading to varying conclusions regarding delimitation of species and their numbers. Rather than disagreeing on the conceptual agreement of what is a species (a separately evolving metapopulation lineage^1^), de Queiroz^2,3^ argues that each species concept emphasizes different properties. In evolutionary biology, “species” are hypotheses for which evidence can be sought by the study of multiple properties^3^. The absence of a certain property does not provide evidence contradicting any given species hypothesis. This is the unifying species concept^3^.

Fungi have essential functions in ecosystems, they are virtually everywhere, even in the most extreme habitats, and associate with diverse organisms (algae, plants, invertebrates and other fungi)^4^. Currently, about 135,000 species of fungi have been described^5^; still many localities, habitats and taxonomic groups remain poorly sampled. In the pre-molecular era, Hawksworth^6^ estimated the number of fungal species to be 1.5 million, based on the ratio of vascular plants and fungi on the British Isles, which he accepted as 1:6. An ITS-based evaluation of soil fungal diversity of two temperate plots and the vascular plant richness in those plots led to an extrapolation of global (soil) fungal species richness estimates from 3.5 to 5.1 million^7^. Taylor and colleagues^8^, using a large fungal dataset from a boreal ecosystem with well-established plant diversity, suggested up to 6 million species of fungi as a global estimate. Understanding how these millions of fungal species have come to existence has stimulated widespread interest. The challenges of diversity studies are posed especially for fungi, which produce propagules that are microscopic in size, have sometimes worldwide distributions and use a multitude of host species. Pringle and colleagues^9^ postulated that morphology is a poor means to distinguish species of this magnitude given these dispersal potentials and patterns of host usage. As a result, species hypotheses about microscopic organisms with global distributions or multiple host ranges should be treated with care.

Many fungal species have been described based on morphological traits; representatives of any given species share a set of morphological characteristics. However, this morphological species concept is a poor means of species delimitation when phenotypic plasticity allows for overlapping morphologies in distinct species or when morphological traits have not yet arisen in the process of speciation or incipient speciation. For example, the genus *Protoparmelia* s. str. (Ascomycota, Lecanorales) consists of 12 species based on morphological and chemical features but a phylogenetic-coalescent approach recognizes 23 species^10^. Another widely cited example is that of *Dictyonema glabratum* (Basidiomycota, Agaricales), a single morphological species that constitutes 126 species using a Generalized Mixed Yule Coalescent (GMYC) analysis of a large dataset of the internal transcribed spacer (ITS) DNA region, and even more than 400 species based on predictive modeling^11^.

Many species of fungi form associations with other organisms and these associations may be critical in species recognition. As a result, fungal species may be circumscribed based on the property of host associations. Host specificity represents an ecological condition; it entails resource availability and niche specialization. The concept of “ecological species” generally refers to reproductive isolation evolved through adaptation to different environments. The micro-evolutionary process of natural selection among diverging populations or subsets of a single population acts in contrasting directions between environments and leads to the fixation of alleles, which may be advantageous in one environment but not in others^12–14^. The ecological species concept dates from the 1940s, when Theodosius Dobzhansky^15^ wrote that “[s]peciation in *Drosophila* proceeds mainly through evolving physiological complexes which are successful each in its environment.” An interesting case is *Leccinum* (Basidiomycota, Boletales), a genus of ectomycorrhizal fungi forming associations with many plant hosts. A recent study^16^ found high host specificity in all species included, except for the generalist *L. aurantiacum*. The authors raised the point that ecological information on its own (“the ability to grow on a new host”) does not a priori provide evidence for a species hypothesis. More recently, three species were described within the ant-parasitic *Ophiocordyceps unilateralis* species complex (Ascomycota, Hypocreales) based on the combination of molecular, micro-morphological and ecological (host specificity) data^17^. All this is in line with de Queiroz’s (2007) view^3^ that multiple properties provide evidence for lineage separation, that is, divergence of populations, and, thus, speciation.

**Figure 1:**
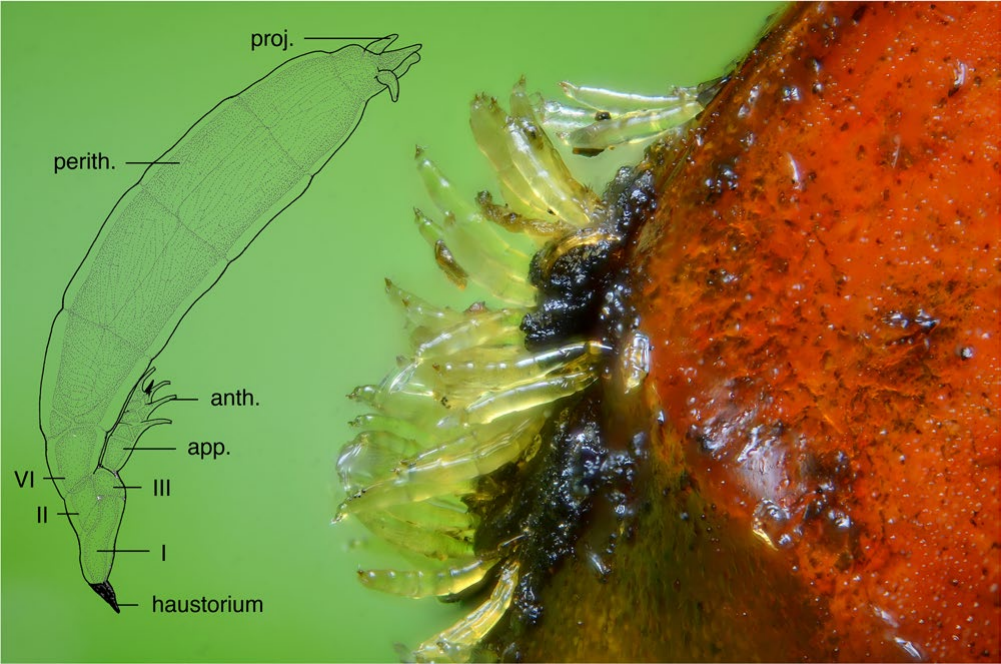
*Hesperomyces virescens* parasitizing *Harmonia axyridis*. A group of thalli of *Hesperomyces virescens* attached to the elytral tips of a specimen of *Harmonia axyridis* (from Watermaal-Bosvoorde, Belgium). Image provided by Gilles San Martin. Inserted is a drawing of a single adult thallus (D. Haelew. 601c, FH 00313615, Byron, Georgia, USA), with the following structures annotated: haustorium; cells I, II, and III of the receptacle; the appendage (app.) with antheridia (anth.); and the perithecium (perith.) with its terminal projections (proj.). Drawing by André De Kesel.

**Figure 2:**
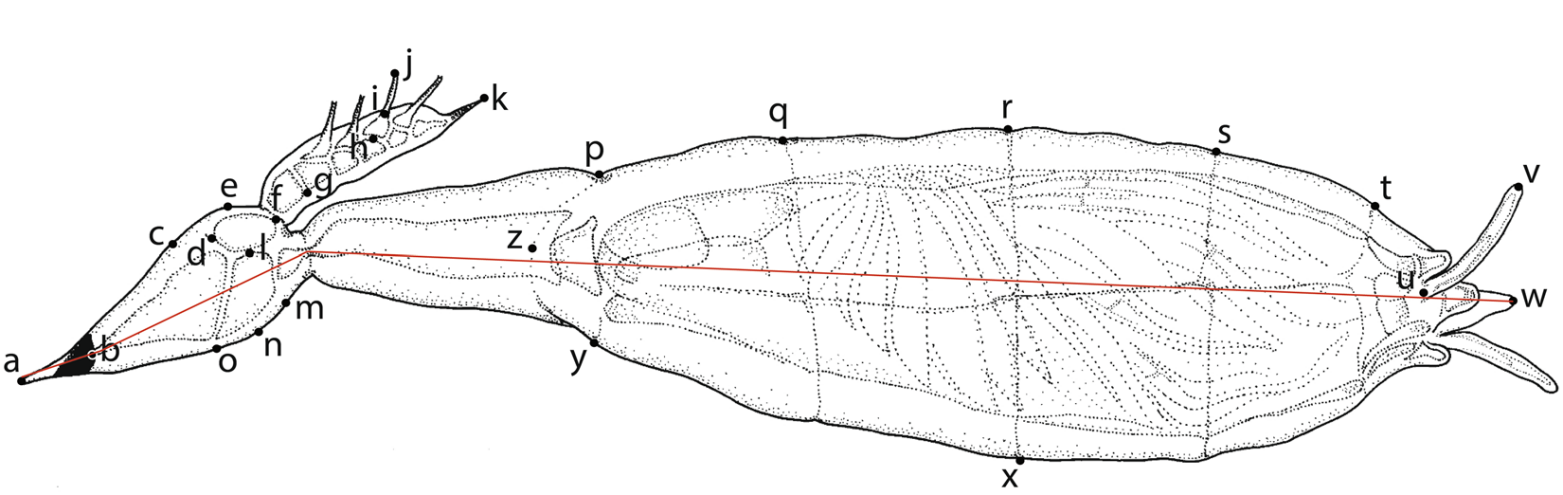
Adult thallus of *Hesperomyces virescens*. Thallus was removed from a specimen of *Psyllobora vigintiduopunctata* (ADK763b, Zwin Nature Park, Belgium). Letters a through z refer to begin and end points (landmarks) for measurements taken of 22 parameters. Details in text.

In this paper, we explore species limits in an enigmatic group of microscopic fungi, the Laboulbeniales. These are obligate, microscopic ectoparasites of arthropods. Around 2200 species in 141 genera are known to infect various groups in three arthropod subphyla (Chelicerata, Hexapoda, Myriapoda) and they are known from all continents except Antarctica^18–21^. Laboulbeniales never form mycelia; the ascospores do not form germ tubes but rather divide mitotically after attachment to the host to form thalli of up to thousands of cells by determinate growth. *Hesperomyces virescens* (Figs 1, 2) is a species of Laboulbeniales that has been reported to parasitize around 30 species of ladybirds (Coleoptera, Coccinellidae) in all continents but Antarctica. It grows exclusively on adult ladybird hosts in 21 genera in 5 subfamilies^21^. Since its discovery on the invasive ladybird *Harmonia axyridis*, biologists have discussed *H. virescens* as a candidate model for studying host-parasite co-evolution and biological control programs^22^. Contrary to what is the consensus for Laboulbeniales, *H. virescens* has been reported to have negative effects on its hosts^21,22^. Based on intra- and interspecific transmission experiments, Cottrell and Riddick^23^ suggested that different lineages of *H. virescens* exist and that each of these lineages may have a high degree of host specificity. The question whether *H. virescens* truly is a single species or an assemblage of morphologically similar but genetically distinct species^19^ has provided the starting point for the present research study. To identify *H. virescens*, mycologists have used morphological characters that can be compared across a range of different host species. Here, we combine morphological, molecular and ecological data as independent lines of evidence to infer the number of species within *H. virescens*, following the unified species concept proposed by de Queiroz^2,3^.

## Results

### Morphometric approach

Details of measurements and ratios for all 181 thalli included in the analysis are given in Supplementary Table S1. For a majority of variables, the best model to explain differences in measurements contained host species (Mod1 in Table 1). Inclusion of host species as an explanatory variable considerably improved model performance. Of the 35 studied variables, 10 did not differ significantly between host species: W cell I, L cell II, W cell II, L cell VI, lngst. proj., L/W cell II, total L rec./total L, tier II/L perith., tier III/L perith., and lngst. proj./L perith.

**Table 1:** Comparison of GLMMs for all variables of *Hesperomyces virescens* thalli removed from different host species.

Variables	Mean	St. dev.	Mod0	Mod1	ΔAIC	Chi-square	*P*
**Measured Parameters**							
total L w foot*	388.24	66.50	1457.4	1448.5	−8.9	12.904	0.0016
total L*	374.33	66.50	1857.9	1849.4	−8.5	12.522	0.0019
L cell I*	59.44	9.66	1103.4	1088.1	−15.3	19.24	0.0000
W cell I	22.14	3.27	901.42	901.22	−0.2	4.1979	0.1226
L cell II^(^*^)^	28.41	5.35	1023.4	1021.5	−1.9	5.8435	0.0538
W cell II	18.45	3.56	884.89	884.41	−0.48	4.4752	0.1067
L cell III*	13.70	3.05	812.53	804.39	−8.14	12.142	0.0023
W cell III*	16.33	3.13	861.48	856.72	−4.76	8.7583	0.0125
total L rec.*	73.79	11.29	1149.8	1138.2	−11.6	15.634	0.0004
L bas. app.*	18.40	2.59	674.38	660.79	−13.59	17.587	0.0002
total L app.*	70.97	7.83	1066.0	1039.2	−26.8	30.768	0.0000
L lngst. anth.*	25.39	2.83	769.99	754.72	−15.27	19.277	0.0000
L anth. neck*	15.18	1.74	621.59	597.66	−23.93	27.93	0.0000
L cell VI	46.91	13.78	1395.6	1396.3	0.7	3.3397	0.1883
W cell VI*	28.43	6.28	1026.5	1018.1	−8.4	12.313	0.0021
L perith.*	262.69	49.57	1733.1	1722.5	−10.6	14.536	0.0007
W perith.*	68.96	10.02	1234.7	1223.8	−10.9	14.901	0.0006
tier II*	65.86	13.91	1325.6	1319.3	−6.3	10.241	0.0060
tier III*	59.97	12.69	1256.3	1245.8	−10.5	14.573	0.0007
tier IV*	40.35	10.14	1159.6	1136.5	−23.1	27.104	0.0000
lobes*	47.62	4.19	905.45	892.02	−13.43	17.431	0.0002
lngst. proj.	31.37	8.21	1200.0	1200.9	0.9	3.0464	0.2180
**Ratios**							
L/W cell I*	2.71	0.45	173.88	156.21*	−17.67	21.67	0.0000
L/W cell II	1.57	0.30	54.933	55.249	0.316	3.6841	0.1585
L/W cell III*	0.85	0.16	−178.84	−189.19	−10.35	14.355	0.0007
total L rec./total L	0.20	0.03	−959.95	−956.78	3.17	0.8295	0.6605
total L app./total L*	0.19	0.04	−829.88	−838.57	−8.69	12.69	0.0018
L/W cell VI*	1.68	0.49	155.81	136.72	−19.09	23.09	0.0000
tier II/L perith.	0.25	0.02	−1002.85	−999.05	3.8	0.2047	0.9027
tier III/L perith.	0.23	0.01	−1056.7	−1056.5	0.2	3.8681	0.1446
tier IV/L perith.*	0.15	0.02	−1014.9	−1043.2	−28.3	32.374	0.0000
lobes/L perith.*	0.19	0.03	−880.54	−885.27	−4.73	8.7339	0.0127
L/W perith.*	3.80	0.40	93.579	82.962	−10.617	14.617	0.0007
L perith./total L*	0.70	0.03	−866.06	−873.16	−7.1	11.091	0.0039
lngst. proj./L perith.	0.12	0.04	−752.50	−750.19	2.31	1.6866	0.4303

ΔAIC is calculated as the AIC for each model with host species as explaining variable (Mod1) minus the AIC of the null model (Mod0). *Significantly different variables among thalli from different host species. ^(^*^)^L cell II is marginally significant (0.05 < *P* < 0.1).

**Figure 3:**
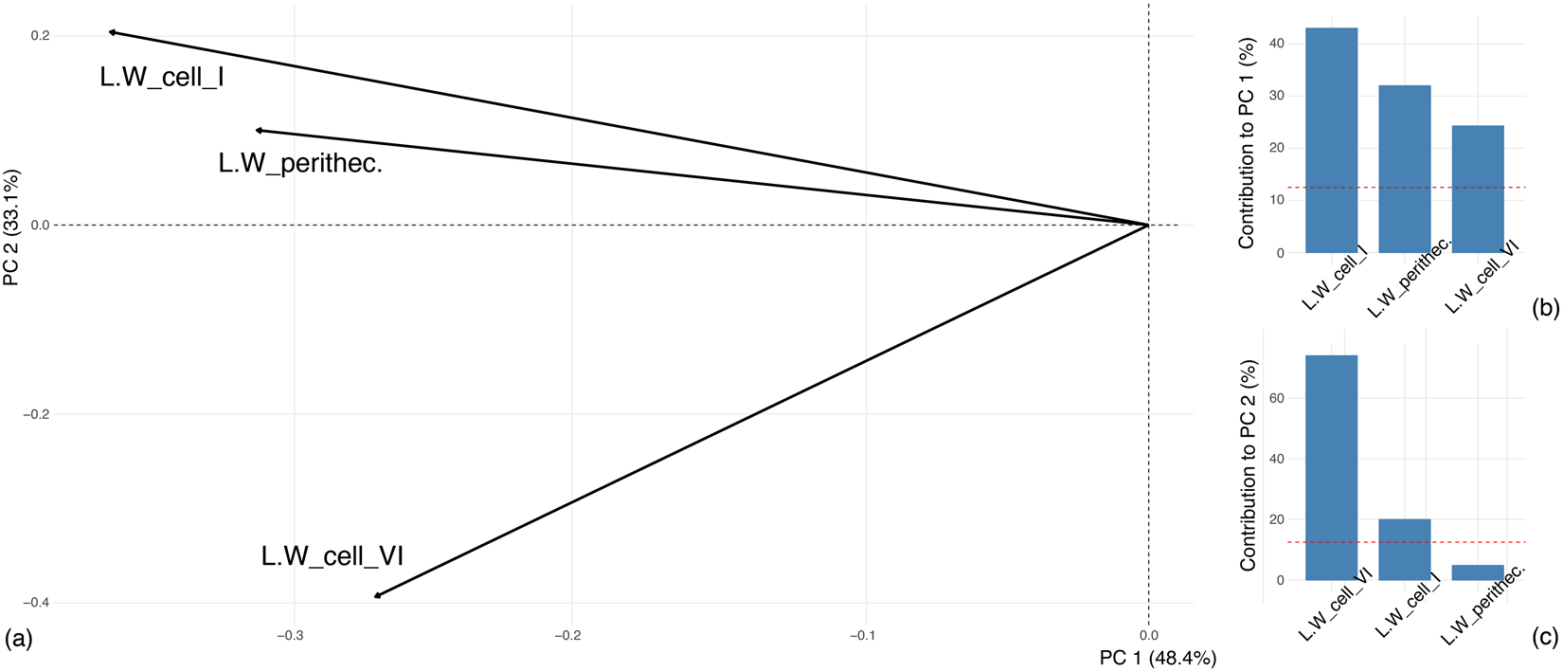
Results of Principal Component Analysis. (**a**) Morphospace formed by the first two PCs of the PCA showing the importance of ratios. (**b**,**c**) Contributions of included ratios to PC1 (**b**) and PC2 (**c**) separately. The dashed line is a reference corresponding to the expected value if the contributions were uniform. Contributions above the reference line are considered as important.

**Figure 4:**
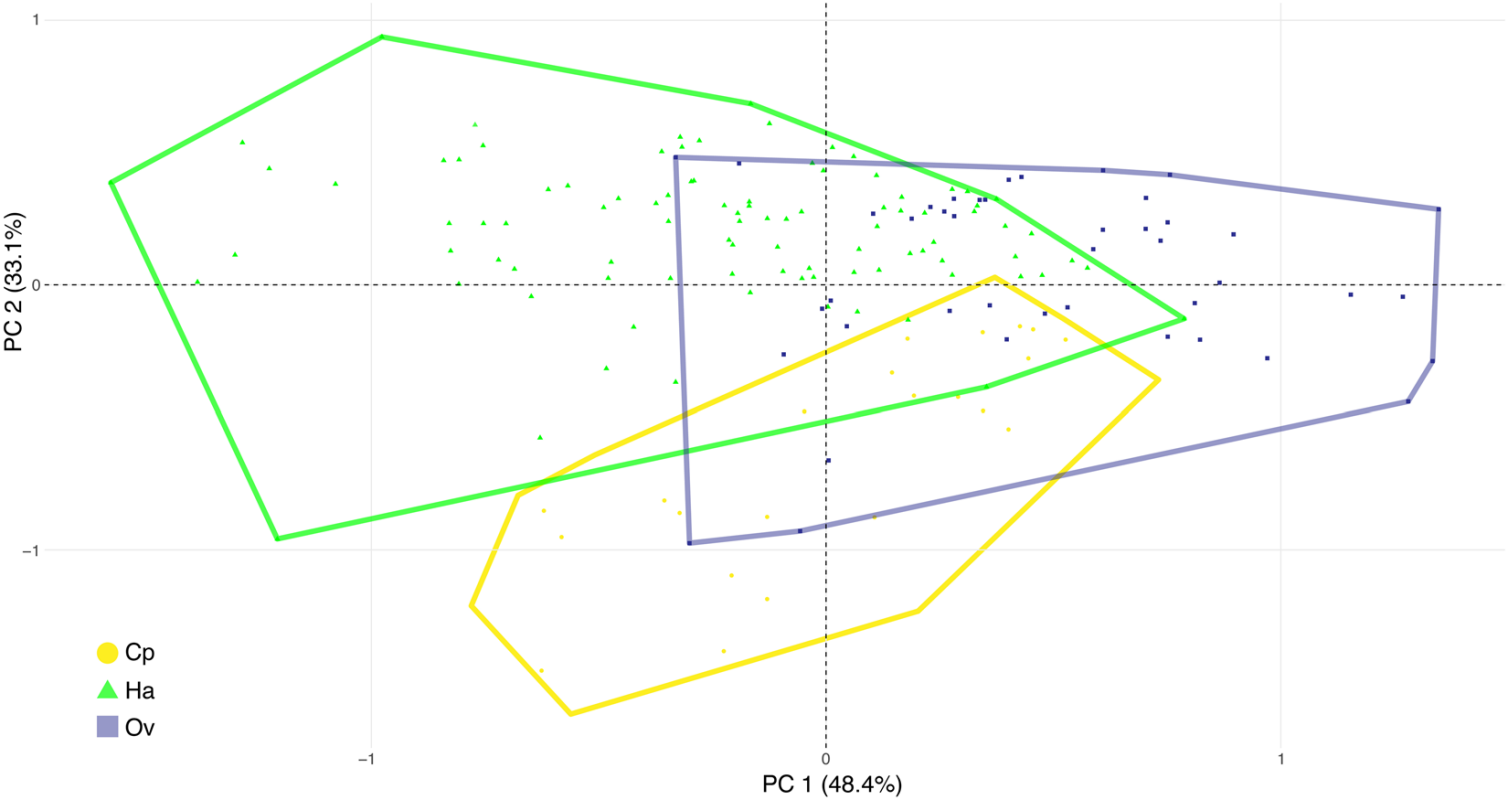
PCA using morphometric variables showing variation in thallus shape. Each symbol represents an individual thallus in the two-dimensional morphospace formed by the first two PCs. Thalli are colored by host species (yellow circles *Cheilomenes propinqua*, green triangles *Harmonia axyridis*, purple squares *Olla v-nigrum*).

We only considered ratios for PCA to focus on shape rather than natural variation in absolute size. Significant differences were observed for the following ratios: L/W cell I, L/W cell III, total L app./total L, L/W cell VI, tier IV/L perith., lobes/L perith., L/W perith., and L perith./total L. Statistical processing of these ratios revealed two principal components (PCs) that together accounted for 81.54% of the observed variation in thallus morphology of *H. virescens* between *C. propinqua*, *H. axyridis* and *O. v-nigrum*. PC1, 48.39% variation explained, represents L/W cell I, L/W perith., and L/W cell VI (Fig. 3a,b). PC2, 33.15% variation explained, represents L/W cell VI and L/W cell I (Fig. 3a,c). In the morphospace formed by the two first PCs, clouds of individuals from the 3 different host species overlap partly, but they also occupy a considerable part of the morphospace without overlap (Fig. 4).

### Nucleotide alignment datasets

We generated 93 *Hesperomyces* sequences during this study, of the SSU (31), ITS (37) and LSU (25) regions. Our ITS dataset comprised 1068 characters, of which 769 were constant and 229 were parsimony-informative. A total of 51 ITS sequences were included, of which 35 have been newly generated during the course of this study, complemented by 16 sequences that we retrieved from GenBank (http://www.ncbi.nlm.nih.gov/genbank/): 43 sequences of *H. virescens*, 2 of *H. coleomegillae*, 2 of *H. palustris* and 4 of *Herpomyces* spp. (outgroup). Isolates of *H. virescens* originated from 9 host species: *Adalia bipunctata* (5 isolates), *A. decempunctata* (2), *Azya orbigera* (1), *Cheilomenes propinqua* (5), *Cycloneda sanguinea* (2), *Halyzia sedecimguttata* (1), *Harmonia axyridis* (16), *Olla v-nigrum* (8) and *Psyllobora vigintimaculata* (3). Our LSU dataset consisted of 22 newly generated sequences of *H. virescens* complemented by 14 sequences downloaded from GenBank (9 of *H. virescens*, 4 of *Herpomyces* spp. and 1 of *Pyxidiophora* cf. *microspora* as outgroup), and 1051 characters, of which 769 were constant and 222 were parsimony-informative. Isolates of *H. virescens* originated from 7 host species: *Adalia bipunctata* (5 isolates), *A. decempunctata* (1), *Azya orbigera* (1), *Cheilomenes propinqua* (2), *Harmonia axyridis* (13), *Olla v-nigrum* (6) and *Psyllobora vigintimaculata* (3).

Our concatenated SSU+ITS+LSU dataset included 2767 characters and 52 isolates representing 6 species (GenBank accession numbers in Table 2). Of all characters, 2183 were constant and 446 were parsimony-informative. Taxonomic sampling covered 3 genera in the Laboulbeniomycetes: *Herpomyces*, *Hesperomyces* and *Pyxidiophora*. In addition to 43 isolates of *Hesperomyces virescens*, we included *Herpomyces chaetophilus, H. periplanetae* and *Pyxidiophora* cf. *microspora* (outgroup). Isolates of *H. virescens* originated from 9 different host species: *Adalia bipunctata* (5 isolates), *A. decempunctata* (2), *Azya orbigera* (1), *Cheilomenes propinqua* (5), *Cycloneda sanguinea* (2), *Halyzia sedecimguttata* (1), *Harmonia axyridis* (16), *Olla v-nigrum* (8) and *Psyllobora vigintimaculata* (3).

**Table 2:** Overview of Laboulbeniomycetes sequences generated and/or used in this study, with indication of datasets in which isolates were used (ITS, LSU, SSU+ITS+LSU).

Species	Isolate, voucher	Geography	Host species	SSU	ITS	LSU	Dataset(s)		
							ITS	LSU	3-gene
**Pyxidiophorales**									
*Pyxidiophora* cf. *microspora*	MG202	Poland		MG438334	MG438314	MG438362		X	X
**Herpomycetales**									
*Herpomyces chaetophilus*	D. Haelew. 483b	USA, Massachusetts	*Periplaneta americana*	MG438319	MG438293	MG438350	X	X	X
*Herpomyces chaetophilus*	D. Haelew. 602b	USA, Massachusetts	*Periplaneta americana*	KT800023	KT800039	KT800009	X	X	X
*Herpomyces periplanetae*	D. Haelew. 602d	USA, Massachusetts	*Periplaneta americana*	MG438327	MG438305	MG438357	X	X	X
*Herpomyces periplanetae*	D. Haelew. 1187d	USA, Massachusetts	*Periplaneta americana*	MG438331	MG438309	MG438359	X	X	X
**Laboulbeniales**									
*Hesperomyces coleomegillae*	631 C	Ecuador	*Coleomegilla maculata*	KF266882	KF192892		X		X
*Hesperomyces coleomegillae*	632 A	Ecuador	*Coleomegilla maculata*	KF266880	KF192888		X		X
*Hesperomyces* aff. *coleomegillae*	D. Haelew. 1287b	Panama	*Coleomegilla maculata*			**MG745334**			
*Hesperomyces* aff. *coleomegillae*	D. Haelew. 1291c	Panama	*Coleomegilla maculata*			**MG745335**			
*Hesperomyces palustris*	631 K	Ecuador	*Coleomegilla maculata*	KF266902	KF192902		X		X
*Hesperomyces palustris*	632B	Ecuador	*Coleomegilla maculata*	KF266891	KF192899		X		X
*Hesperomyces* aff. *palustris*	D. Haelew. 1325a	Panama	*Coleomegilla maculata*		**MG745336**	**MG745336**			
*Hesperomyces virescens*	D. Haelew. 316a	USA, Georgia	*Harmonia axyridis*	MG438339	MG438315	KJ842339	X	X	X
*Hesperomyces virescens*	D. Haelew. 334b	Netherlands	*Harmonia axyridis*	MG438340	MG438316	MG438364	X	X	X
*Hesperomyces virescens*	JP352b	USA, Georgia	*Olla v-nigrum*	**MG760581**	**MG757798**	**MG745337**	X	X	X
*Hesperomyces virescens*	JP353a	USA, Georgia	*Olla v-nigrum*	KT800028	KT800043	KT800013	X	X	X
*Hesperomyces virescens*	JP353b	USA, Georgia	*Olla v-nigrum*	**MG760582**	**MG757799**	**MG745338**	X	X	X
*Hesperomyces virescens*	JP354b	USA, Georgia	*Olla v-nigrum*	**MG760583**	**MG757800**	**MG745339**	X	X	X
*Hesperomyces virescens*	D. Haelew. 361a	Netherlands	*Harmonia axyridis*	**MG760584**	**MG757801**		X		X
*Hesperomyces virescens*	D. Haelew. 486c	USA, Massachusetts	*Harmonia axyridis*	**MG760585**	KT800044	KT800014	X	X	X
*Hesperomyces virescens*	HM497c	USA, Georgia	*Harmonia axyridis*	KT800030	KT800046	KT800016	X	X	X
*Hesperomyces virescens*	D. Haelew. 516a	USA, Massachusetts	*Harmonia axyridis*	**MG760586**	**MG757802**	**MG745340**	X	X	X
*Hesperomyces virescens*	D. Haelew. 646a	Germany	*Harmonia axyridis*	**MG760587**	**MG745341**	**MG745341**	X	X	X
*Hesperomyces virescens*	D. Haelew. 646c	Germany	*Harmonia axyridis*	**MG760588**	KT800045	KT800015	X	X	X
*Hesperomyces virescens*	D. Haelew. 648c	South Africa	*Harmonia axyridis*	KU574863	KU574864	KU574865	X	X	X
*Hesperomyces virescens*	D. Haelew. 653a	South Africa	*Cheilomenes propinqua*	**MG760589**	**MG757803**		X		X
*Hesperomyces virescens*	D. Haelew. 655c	South Africa	*Cheilomenes propinqua*	KU574866	**MG757804**	KU574867	X	X	X
*Hesperomyces virescens*	D. Haelew. 659b	South Africa	*Cheilomenes propinqua*	**MG760590 (659a)**	**MG757805**	**MG745342**	X	X	X
*Hesperomyces virescens*	D. Haelew. 659d	South Africa	*Cheilomenes propinqua*	**MG760591**	**MG757806**		X		X
*Hesperomyces virescens*	D. Haelew. 669a	South Africa	*Harmonia axyridis*		**MG757807**				
*Hesperomyces virescens*	D. Haelew. 924a	Panama	*Cycloneda sanguinea*	KX533512 (929a)	**MG757808**		X		X
*Hesperomyces virescens*	D. Haelew. 928g	Panama	*Azya orbigera*	**MG760592**	**MG745343**	**MG745343**	X	X	X
*Hesperomyces virescens*	D. Haelew. 943a	South Africa	*Harmonia axyridis*	**MG760593**	**MG757809**		X		X
*Hesperomyces virescens*	D. Haelew. 943b	South Africa	*Harmonia axyridis*	**MG760594**	**MG757810**	**MG745344**	X	X	X
*Hesperomyces virescens*	D. Haelew. 954d	USA, Georgia	*Olla v-nigrum*	**MG760595**	**MG757811**		X		X
*Hesperomyces virescens*	D. Haelew. 954e	USA, Georgia	*Olla v-nigrum*	**MG760596**	**MG757812**		X		X
*Hesperomyces virescens*	D. Haelew. 955b	Netherlands	*Halyzia sedecimguttata*		**MG757813**		X		X
*Hesperomyces virescens*	D. Haelew. 1005c	South Africa	*Harmonia axyridis*	**MG760597**	**MG757814**		X		X
*Hesperomyces virescens*	D. Haelew. 1174a	Netherlands	*Harmonia axyridis*	**MG760598**	**MG757815**	**MG745345**	X	X	X
*Hesperomyces virescens*	D. Haelew. 1188g	USA, Massachusetts	*Harmonia axyridis*	MG438341	MG438317	MG438365	X	X	X
*Hesperomyces virescens*	D. Haelew. 1193a	Denmark	*Adalia bipunctata*		**MG757816**				
*Hesperomyces virescens*	D. Haelew. 1193g	Denmark	*Adalia bipunctata*	**MG760599**	**MG757817**	**MG745346**	X	X	X
*Hesperomyces virescens*	D. Haelew. 1199h	Sweden	*Adalia bipunctata*	**MG760600**	**MG757818**	**MG745347**	X	X	X
*Hesperomyces virescens*	D. Haelew. 1200h	USA, Georgia	*Olla v-nigrum*	**MG760601**	**MG757819**	**MG745348**	X	X	X
*Hesperomyces virescens*	D. Haelew. 1200i	USA, Georgia	*Olla v-nigrum*	**MG760602**	**MG757820**	**MG745349**	X	X	X
*Hesperomyces virescens*	D. Haelew. 1231a	Italy	*Adalia bipunctata*	**MG760603**	**MG757821**	**MG745350**	X	X	X
*Hesperomyces virescens*	D. Haelew. 1232a	Italy	*Adalia bipunctata*	**MG760604**	**MG757822**	**MG745351**	X	X	X
*Hesperomyces virescens*	D. Haelew. 1247a	Italy	*Adalia bipunctata*	**MG760605**	**MG745352**	**MG745352**	X	X	X
*Hesperomyces virescens*	D. Haelew. 1248b	Italy	*Adalia decempunctata*	**MG760606**	**MG757823**	**MG745353**	X	X	X
*Hesperomyces virescens*	D. Haelew. 1249a	Italy	*Adalia decempunctata*		**MG757824**		X		X
*Hesperomyces virescens*	D. Haelew. 1250b	USA, California	*Psyllobora vigintimaculata*	**MG760607**	**MG757825**	**MG745354**	X	X	X
*Hesperomyces virescens*	D. Haelew. 1250c	USA, California	*Psyllobora vigintimaculata*	**MG760608**	**MG757826**	**MG745355**	X	X	X
*Hesperomyces virescens*	D. Haelew. 1251b	USA, California	*Psyllobora vigintimaculata*	**MG760609**	**MG757827**	**MG745356**	X	X	X
*Hesperomyces virescens*	D. Haelew. 1259a	South Africa	*Cheilomenes propinqua*		**MG757828**		X		X
*Hesperomyces virescens*	D. Haelew. 1268b	Japan	*Harmonia axyridis*	**MG760610**	**MG757829**	**MG745357**	X	X	X
*Hesperomyces virescens*	D. Haelew. 1268d	Japan	*Harmonia axyridis*	**MG760611**	**MG757830**	**MG745358**	X	X	X
*Hesperomyces virescens*	D. Haelew. 1374a	Panama	*Cycloneda sanguinea*		**MG757831**		X		X

All isolates of which sequences were generated are listed, with country and host species information as well as GenBank accession numbers. Sequences in bold were generated during the course of this study.

### Phylogenetic inferences

The *Hesperomyces* clade has maximum support in the ITS and LSU datasets. In both datasets, each monophyletic clade within the *H. virescens* complex consists of isolates from thalli removed from a single host species. There is one exception, the clade consisting of isolates from two host species in the same genus, *Adalia bipunctata* and *A. decempunctata*. Henceforward, in the text we will refer to these distinct clades by the first letters of the host genus and species names: Ab for *Adalia bipunctata*, Ad for *Adalia decempunctata*, Ao for *Azya orbigera*, Cp for *Cheilomenes propinqua*, Cs for *Cycloneda sanguinea*, Ha for *Harmonia axyridis*, Hs for *Halyzia sedecimguttata*, Ov for *Olla v-nigrum* and Pv for *Psyllobora vigintimaculata*. In addition, figures are consistently color-coded by host species where appropriate.

**Figure 5:**
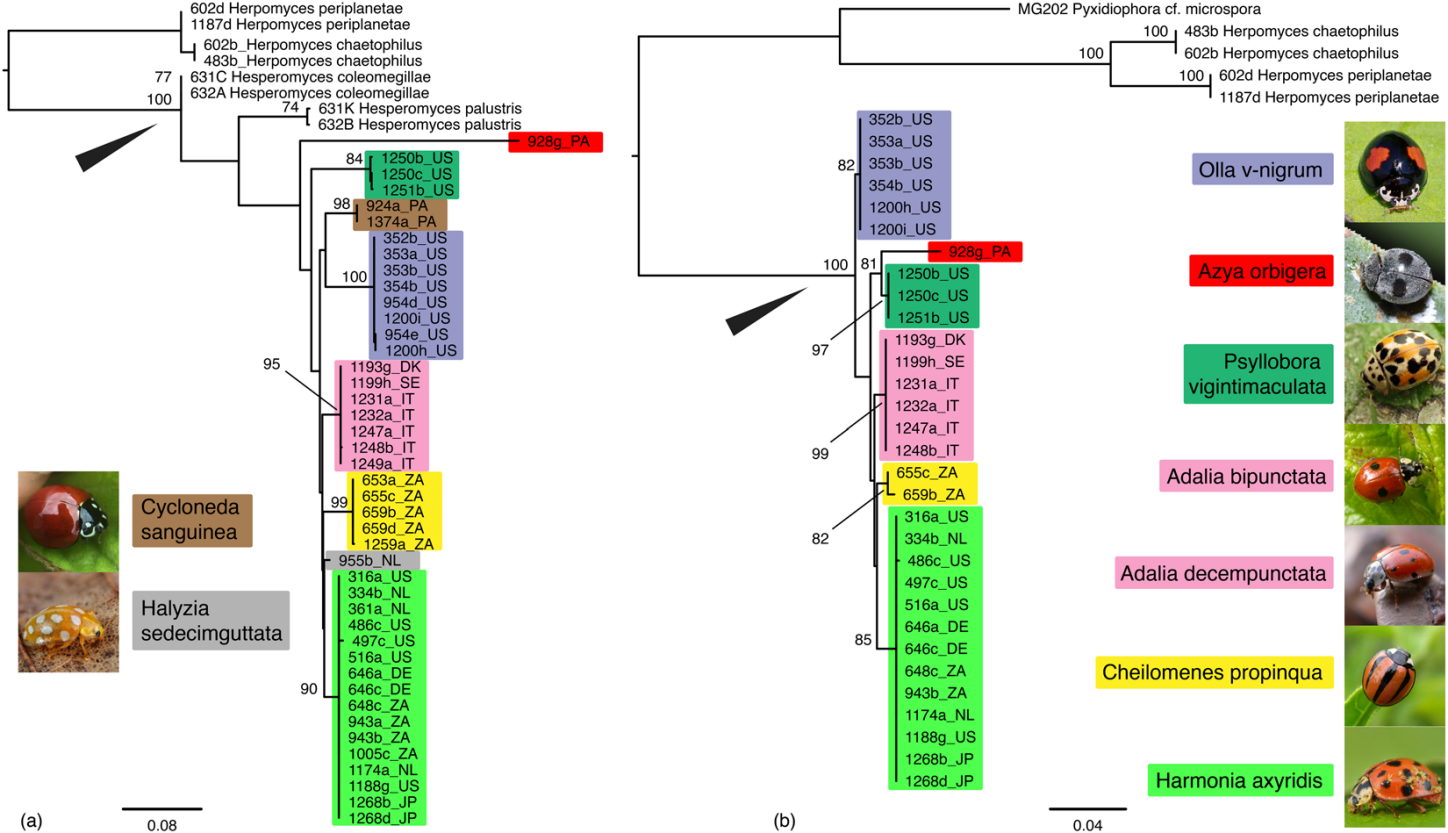
Phylogenetic reconstruction of *Hesperomyces virescens* sensu lato. (**a**) Phylogram reconstructed from the ITS dataset. (**b**) Phylogram reconstructed from the LSU dataset. Topologies are the result of maximum likelihood inference. For each node, the ML bootstraps (if >70) are presented above the branch leading to that node. The arrowhead in both reconstructions points at the node leading to the *Hesperomyces* clade. Monophyletic clades are color-coded by host species. All host species included in the analysis are pictured left and right of the phylogenies. Photo credits: *Adalia bipunctata*, *A. decempunctata*, *Halyzia sedecimguttata*, *Harmonia axyridis*, Gilles San Martin (Flickr); *Azya orbigera*, Pavel Kirillov (Wikimedia Commons); *Cheilomenes propinqua*, Sally Adam (iNaturalist); *Cycloneda sanguinea*, Damon Tighe (iNaturalist); *Olla v-nigrum*, Roberto Güller (Flickr); *Psyllobora vigintimaculata*, Katja Schulz (iNaturalist).

**Figure 6:**
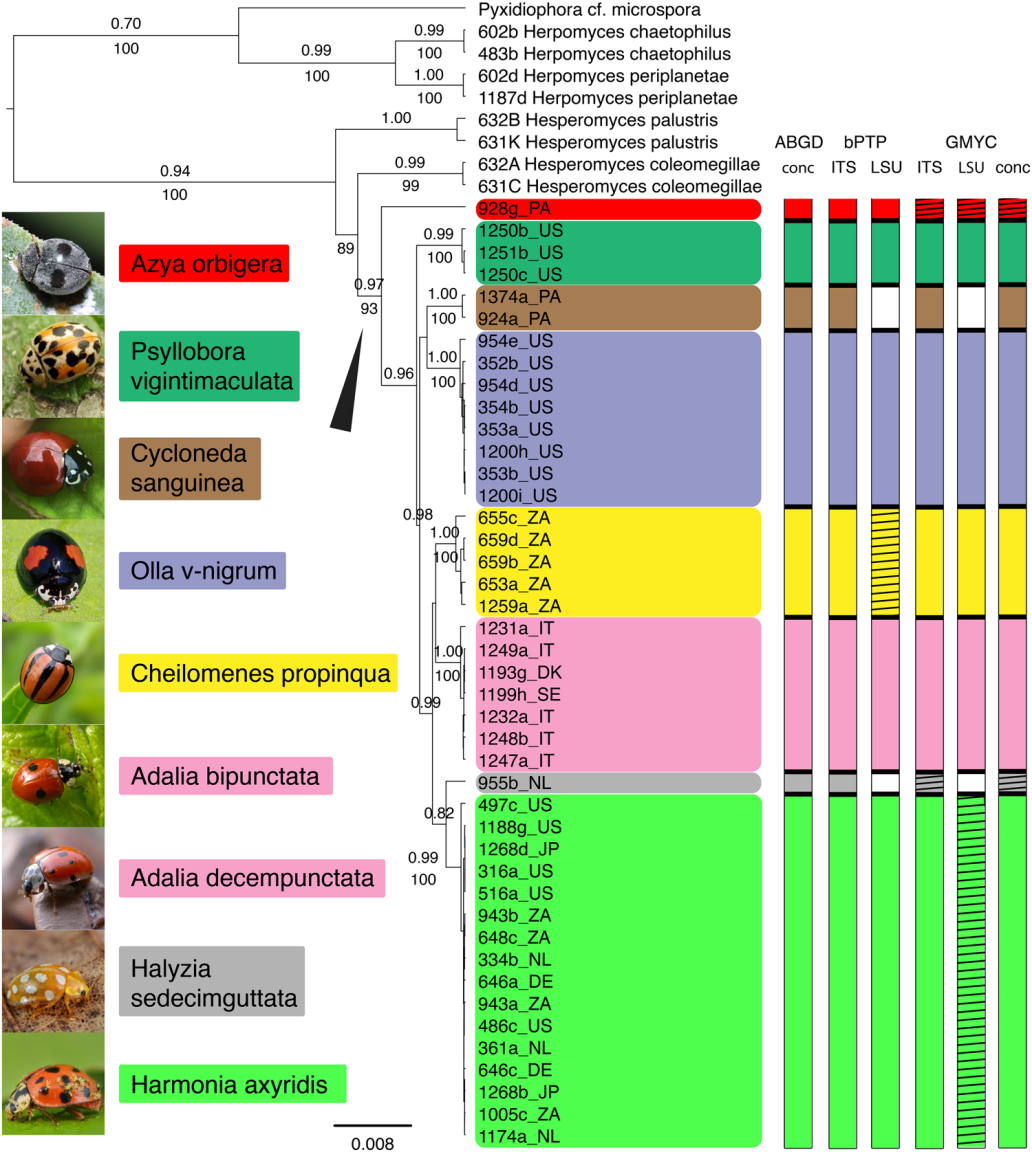
Three-gene phylogeny showing results of sequence-based species delimitation methods. Maximum clade credibility tree of the *Hesperomyces virescens* complex, reconstructed from the concatenated SSU+ITS+LSU dataset. The tree is the result of a Bayesian analysis performed in BEAST. For each node, the ML bootstraps (if >70) and posterior probabilities (if >0.70) are presented above/below the branch leading to that node. Monophyletic clades are color-coded by host species. To the left of the phylogeny, all host species included in the analysis are pictured. Photo credits: *Adalia bipunctata*, *A. decempunctata*, *Halyzia sedecimguttata*, *Harmonia axyridis*, Gilles San Martin (Flickr); *Azya orbigera*, Pavel Kirillov (Wikimedia Commons); *Cheilomenes propinqua*, Sally Adam (iNaturalist); *Cycloneda sanguinea*, Damon Tighe (iNaturalist); *Olla v-nigrum*, Roberto Güller (Flickr); *Psyllobora vigintimaculata*, Katja Schulz (iNaturalist). To the right of the phylogeny, the results of species delimitation analyses are summarized, from left to right: ABGD analysis of the SSU+ITS+LSU alignment; bPTP analysis of the ITS topology; bPTP analysis of the LSU topology; and GMYC analysis of the ITS, LSU and SSU+ITS+LSU ultrametric trees (without outgroups) generated in BEAST. Hatching implies lack of support, whereas no coloration means that clade was absent in that analysis.

In the ITS dataset, 8 clades are recognized, in addition to *H. coleomegillae* and *H. palustris*, which are positioned basally compared to the other clades (Fig. 5a). Of the 8 clades, 6 are strongly supported (BS ≥ 84): Ab+Ad, Cp, Cs, Ha, Ov and Pv. Only the singleton clades (Ao and Hs) lack support. In the LSU dataset, 6 clades are recognized (Fig. 5b). All of these clades have strong support (BS ≥ 81). In the three-gene dataset, again, the *Hesperomyces* clade has strong support and consists of 10 clades (Fig. 6). Of these, nine have high support (ML BS ≥ 93, pp ≥ 0.94). Only the Hs clade is unsupported in the ML analysis (BS = 65) whereas the Bayesian analysis provides moderate support (pp = 0.82). This clade consists of only a single isolate for which we were able to generate a 784-bp ITS sequence only. ML inference agrees fully with the Bayesian analysis. In both reconstructions, *H. coleomegillae* and *H. palustris* are placed basally compared to the other clades within the genus *Hesperomyces*.

### Species delimitation analyses

Results of the sequence-based methods for species delimitation are summarized in Fig. 6 and Table 3. The ABGD analysis resulted in 8 distinct groups, irrespective of distance metrics or gap width values. Eight species were identified within the *Hesperomyces virescens* clade from the bPTP analysis of the ITS topology. The bPTP analysis of the LSU topology resulted in 6 species (no LSU sequences were generated for isolates from *Cycloneda sanguinea* and *Halyzia sedecimguttata*). Support was lacking for the Cp clade in this analysis. The GMYC analyses of the ITS and concatenated trees resulted in 8 species, all with high support except for clades Ao and Hs (which comprised a single isolate only). The GMYC analysis of the LSU tree resulted in 6 recognized species, without support for Ao, Hs (each comprising a single isolate), and Cp and Ha.

**Table 3:** Summary of results of ML, Bayesian and species delimitation analyses (ABGD, bPTP, GMYC).

Putative species	ML BS ITS	ML BS LSU	ML BS 3 genes	pp 3 genes	ABGD 3 genes	bPTP ITS	bPTP LSU	bPTP 3 genes	GMYC ITS	GMYC LSU	GMYC 3 genes
Ao clade	—	81	93	0.97	+	1.00	1.00	1.00	0.00	0.02	0.00
Pv clade	84	97	100	0.99	+	0.92	0.84	0.34	1.00	0.77	1.00
Cs clade	98		100	1.00	+	0.99		0.49	1.00		1.00
Ov clade	100	82	100	1.00	+	0.98	0.83	0.20	1.00	0.69	1.00
Cp clade	99	82	100	1.00	+	0.99	0.00	0.52	1.00	0.18	1.00
Ab+Ad clade	95	99	100	1.00	+	0.72	0.71	0.20	1.00	0.61	1.00
Hs clade	—		—	0.82	+	1.00		0.99	0.00		0.00
Ha clade	90	85	100	0.99	+	0.91	0.56	0.02	1.00	0 0.41	0.67

Explanation of symbols and values used: — indicates no support; no value indicates that a clade was absent from the analysis; + under ABGD represents supported clades; numbers under bPTP are Bayesian support values for the delimited clades.

## Discussion

Our results illustrate that *H. virescens* encompasses several unique genetic lineages. Each of these lineages occurs on a single host species (or two host species in the case of *Adalia*), regardless of geographic origin of the collection. Some of the clades in our phylogenies have no or only moderate support but these are the clades for which only a single isolate is available (Ao and Hs). Some ladybird species were only recently discovered as hosts and others are not frequently found. For example, during fieldwork in Panama in 2015 we found *Hesperomyces* thalli on *Azya orbigera*, which had previously not been reported as a host. Out of 151 ladybirds, only 10 infected ones were found, each individual carrying a single thallus (1 individual carried 3 thalli). We tried two extraction protocols, each with 1 thallus. The extraction using the Extract-N-Amp PCR Plant Kit failed; the one with the REPLI-g Single Cell Kit was successful and we were able to generate SSU, ITS and LSU sequences (isolate D. Haelew. 928 g). In another case, a single *Hesperomyces*-infected individual of *Halyzia sedecimguttata* was found in the Netherlands in 2015. It bore 13 adult and 4 juvenile thalli. We chose to use 10 adult thalli for DNA isolation with the Extract-N-Amp Plant PCR Kit (isolate D. Haelew. 955b) but for this isolate we were only able to generate an ITS sequence. Since this report, no further infected specimens of *H. sedecimguttata* have been collected. Finally, we only had 3 infected individuals of *Psyllobora vigintimaculata* available for study, but these specimens carried sufficient thalli for both morphological study and molecular work.

Some of our host species were collected only from a single population. This is the case for *Cheilomenes propinqua*, *Olla v-nigrum* and *P. vigintimaculata*. However, specimens of *O. v-nigrum*, although from a single locality, were collected on multiple occasions in 2014, 2015 and 2016 (also from a laboratory colony for many generations). *Adalia bipunctata* and *H. axyridis* are the host species with the widest geographical range included in this study. Infected specimens of *A. bipunctata* were collected in Denmark, Italy and Sweden; specimens of *H. axyridis* were collected on different continents. Even so, both clades Ab+Ad and Ha each form two monophyletic lineages, in all datasets (ITS, LSU, SSU+ITS+LSU). In other words, there is no geographic signal. We conclude that phylogenetic structure is primarily determined by host specialization. Based on intra- and interspecific transmission experiments, Cottrell and Riddick^23^ proposed that “isolates/strains of *H. virescens* may exist under field conditions and only infect closely related Coccinellidae or even a single species.” Based on the results of our species delimitations analyses, we propose that these lineages (or clades, as we refer to them) represent distinct species.

The clade Ab+Ad is the only clade consisting of *H. virescens* isolates taken from more than a single host species. In this case, the isolates originated from either *Adalia bipunctata* or *A. decempunctata*. Ladybirds in the genus *Adalia* are popular for studies in ecology and population genetics because they vary distinctively in their elytral and pronotum color patterns. Each of these color forms, or phenotypes, corresponds to a unique genotype^24^. *Adalia bipunctata* and *A. decempunctata* are very similar in ecology and habitat use; both coexist throughout temperate regions in Europe. They are also closely related genetically, and even some *A. bipunctata* × *A. decempunctata* hybrids have been reported^25,26^. This Ab+Ad clade makes for an interesting case because one could pose the question whether the specificity of *H. virescens* relates to host species-level or genus-level. Based on the currently available data we cannot solve this question, but we will continue to collect ladybirds and thus increase our dataset of ladybird host species.

### Comparison of species delimitation methods

Whereas molecular data provide a valuable tool to validate morphology-based species descriptions, the application of species delimitation methods can increase confidence if several methods offer congruent estimates of species diversity within a given dataset. Incongruences in results imply that multiple methods differ in their delimitation power. Alternatively, it is also possible that users make incorrect assumptions when employing a given species delimitation approach^27^. In the event of incongruent results, it is better to be conservative, rather than to designate entities that do not actually represent evolutionary metapopulation lineages as species. In any case, the multiple species delimitation analyses that we used in our study identified congruent species boundaries. The combination of BS, pp and species delimitation support provides strong evidence for *H. virescens* being a complex of multiple species.

In the bPTP analysis of the LSU topology, the Cp clade was not supported. PTP models speciation in terms of number of nucleotide substitutions^28^. Upon manual inspection of the multiple alignment, for all clades it is the case that the number of nucleotide substitutions within the clade is zero. The only exception is the Cp clade, with 2 substitutions between the isolates of this clade. The number of substitutions between Cp and other clades ranges from 5 to 13. The PTP model did probably not interpret the Cp clade as a distinct species because of these *within-clade* substitutions for the Cp clade. Our PTP analyses based on single-gene trees are consistent with the results obtained by ABGD. We also performed a bPTP analysis on the concatenated SSU+ITS+LSU dataset (only Bayesian support values shown, Table 3). Although the number of species is the same as in the PTP analysis of single gene topology and the ABGD and GMYC approaches, the Bayesian support dropped significantly; none of the delimited clades have support higher than 0.52. We repeat earlier findings^28,29^ that PTP is most accurate with single gene trees.

Kekkonen and Hebert^30^ put forward that GMYC usually delimits more species compared to other methods. In our analysis, the results from GMYC are congruent with ABGD and bPTP of the ITS topology (and bPTP of the LSU topology, noting that two clades were missing in this analysis). Two clades that lack support – Ao and Hs – consist of single isolates only. GMYC looks at intraspecific branching versus interspecific branching, and thus it is no surprise that these singleton clades have no support from this approach. The low support for the Ha clade in the GMYC analyses of the LSU and three-gene topologies may be explained by the fact that for a number of isolates coming from *Harmonia axyridis*, sequences are incomplete (missing or only partial SSU, ITS or LSU). Because this clade holds many isolates, missing sequence data for a number of these isolates may influence generating an ultrametric tree, which is a computationally intensive and error-prone process. Since GMYC is dependent on the accuracy of this input tree, any alterations will strongly influence species delimitation analyses.

Upon the introduction of PTP, it was noted that delimited groups represent “putative” species^28^. PTP uses phylogenetic reconstructions inferred from single gene datasets, which are gene trees rather than species trees. Also GMYC is based on a single-gene tree. As a consequence, more data should be collected to confirm and validate the species boundaries set by these delimitation approaches, in an integrative taxonomy framework across disciplines^31–35^. Note that this framework is in line with the unified species concept^3^.

### Comparison of ITS and LSU as barcode markers

Molecular identification of fungi relies on the availability of good DNA barcode markers. Currently, DNA-based identifications focus on genes that code for ribosomal RNA (rDNA), because these regions have many copies in the genome and thus are well-suited target regions for PCR amplification. The internal transcribed spacer (ITS) region has been proposed as universal barcode for Fungi^36^. This means that for a majority of fungi, the interspecific variation at this marker should exceed the intraspecific variation, and for over 70% of fungi the ITS is indeed effective in recognizing species. A number of considerations have been made since the acceptance of this barcode marker^36–39^: (1) RPB1 is actually better in discriminating species but its amplification success is much lower; (2) whereas ITS performs best overall across the fungal kingdom, its identification power is equal to LSU for subphyla Pezizomycotina and Saccharomycotina (Ascomycota); (3) ITS does not contain enough variation to discriminate between species for some groups of fungi, such as *Aspergillus* and economically important plant pathogens in the genera *Alternaria*, *Diaporthe*, *Fusarium* and others; (4) arbuscular mycorrhizal-forming species in Glomeromycota are multinucleate and extremely intraspecific divergent in their ribosomal DNA. These challenges have driven mycologists to developing other, lineage- or genus-specific barcodes. These secondary barcodes are often more difficult to amplify (because of the lack of universal primers) but have a better delimiting power than the ITS^40^. Examples of secondary barcodes include calmodulin (CaM) for *Aspergillus*; the translation elongation factor (*TEF1*), topoisomerase I (*TOP1*) and phosphoglycerate kinase (*PGK*) for *Fusarium*; the LSU rDNA region for *Amanita*; and the Apn2-Mat1–2 intergenic spacer and partial mating type (Mat1–2) gene (*ApMat*) and glutamine synthetase (*GS*) combined for *Colletotrichum*^38,41,42^.

We experience low amplification success for the ITS region with the Laboulbeniales using general fungal-specific primers. There are many possible reasons for failed ITS amplification, ranging from simple primer mismatch as is the case in the Archaeorhizomycetes^43^ to significant intragenomic^44,45^ or intraspecific variability^46^. Although the 5.8S region is highly conserved, both spacer regions (especially ITS1) appear to be rapidly evolving^47^. Previously generated sequences of Laboulbeniales suggest that the ITS differs significantly among genera and we have currently no idea of the extent of this variability. Still, ITS may be useful and important as a marker to study intrageneric relationships. During our studies of *Hesperomyces* we designed and are routinely using primers that specifically target conserved regions of the ITS (ITShespL, 5′-CTCCTGTAGAACCTACACATC-3′ and ITShespR, 5′-CAAATTTAAGCTTTTGCCGC-3′).

During the course of this study, we constructed phylogenies based on single genes (SSU, ITS, LSU) and on a concatenated SSU+ITS+LSU dataset. The SSU gene is very conservative and has no discriminative power at the species level. But, both the ITS and LSU datasets result in high support for the individual clades of the *H. virescens* complex (Fig. 6). In addition to its discrimination power, amplification of the LSU region poses virtually no problem within the Laboulbeniomycetes so far investigated. The commonly used fungal primers for the LSU region, such as LR0R/LR5 and LIC24R/LR3^48,49^, generally work well for most species of Laboulbeniomycetes. Based on these preliminary results, the LSU region should be further investigated as barcode for species delimitation in Laboulbeniomycetes.

### Hesperomyces virescens, a complex of cryptic species?

Recent molecular (phylogenetic) studies point at a dazzling diversity of the kingdom Fungi. However, it is not always possible to infer this diversity from morphological features. Species that “have been classified as a single nominal species because they are at least superficially morphologically indistinguishable” are referred to as cryptic species^50^. Many (or almost any^51^) species studied using molecular, incompatibility, secondary metabolites have been shown to “mask” several biological species. Examples are found in diverse groups of fungi – among Ascomycota: Eurotiales (*Aspergillus*^9^), Helotiales (*Phialocephala*^52^), Lecanorales (*Protoparmelia*^10^); among Basidiomycota: Agaricales (*Cortinarius*^53^; *Tricholomopsis*^54^), Polyporales (*Ganoderma*^55^), Russulales (*Lactifluus*^56^, *Russula*^57^), Ustilaginales (*Tranzscheliella*^58^). In this study, we have observed at a superficial level cryptic species. Consequently, we employed landmark-based geometric morphometry^59^ followed by principal component analysis of shape variation, aimed at finding characters, if any, to circumscribe species with.

To date, morphometric methods in Laboulbeniales have only been applied in studies dealing with the genus *Laboulbenia*. Morphological plasticity of *L. flagellata* from different carabid hosts (Coleoptera, Carabidae) was studied by De Kesel and Van Den Neucker^60^. The general habitus of thalli was stable, but size was related to host species, habitat of the host and position of the fungus on the host integument. Subsequently, De Kesel and Haelewaters^61^ tested differences in thallus shape and dimension between two morphologically similar species of *Laboulbenia*. Most variables were significantly different between both species, particularly the shape of the receptacle was different regardless of size or growth position. In this study, we generated the largest morphometric dataset to date for any species of Laboulbeniales, including measurements and ratios of 181 thalli from 3 host species. Our PCA suggests that the shapes of cell I, cell VI and the perithecium contribute most to the observed variation within the dataset. If we were to formally describe clades Cp, Ha and Ov as separate species, we expect to find most descriptive features in cell I of the receptacle and in the perithecium and its basal cell (VI).

In both the ITS and three-gene phylogenies, we retrieved *H. coleomegillae* and *H. palustris*^62^ (on *Coleomegilla maculata*) basal to the *H. virescens* clade. The basal-most clade within *H. virescens* is Ao in the ITS and three-gene analysis. In terms of morphology, the thalli on *Azya orbigera* are structurally quite different compared to thalli from other host species. For example, the appendage is only 3-celled with the third cell carrying two antheridia. This structure is completely different than thalli from any of the other hosts in our dataset and also than *H. coleomegillae* and *H. palustris*^21,62–64^. The appendage of these thalli consists of a single row of at least 4 cells, and every cell starting from the second carries an antheridium (the distal-most cell carries 2 antheridia). In the LSU phylogeny, the Ov clade is basal-most positioned. However, this tree lacks internal support, except for the sister relationship between clades Ao and Pv. Also the ITS phylogeny lacks internal support and all internal clades collapse to a basal polytomy. On the other hand, incongruence between trees is possible as a result of incomplete lineage sorting. Gene trees are not equal to species trees; a multi-locus approach is preferred for investigating species divergence and gauging relationships between species^65^. In the three-gene phylogeny, the backbone relationships within the *H. virescens* clade are well resolved, as indicated by high pp values. The sister relationship between clades Cs and Ov is without support, as is that between clades Ab+Ad and Cp. This may indicate that there is a lack of taxon sampling, which is no surprise given the broad host range of *H. virescens*^21^. Given the available data, we accept the clades Ab+Ad, Ao, Cp, Cs, Ha, Hs, Ov and Pv to be part of the *H. virescens* complex, or *H. virescens* sensu lato, and to represent independent evolutionary lineages. Following the strict host specificity detected by this study, we propose to restrict *H. virescens* sensu stricto to those thalli found on *Chilocorus stigma*, the host species on which the fungus was originally described^64^.

### Conclusions

Through DNA isolation, PCR amplification, sequencing and analysis methods, thousands of characters became available for minute fungi that do not have many morphological features and do not grow in culture. These remarkable improvements in the collection of character data will help us answer questions about the validity of “worldwide” and “cosmopolitan” geographic distributions ascribed to many morphological forms of the Laboulbeniomycetes. Here, we provided answers in the case of *Hesperomyces virescens*, which we have shown to be a complex of different species, each with its own host (genus). We are only starting to unravel patterns of speciation in this group of fungi. The findings of this paper are not only promising for future studies, but they also emphasize the necessity for an integrative approach in taxonomic research. We hope with this contribution to include the Laboulbeniales ectoparasitic fungi in contemporary discussions considering molecular evolution and speciation patterns, rather than treating them as obscure fungi for specialists only.

## Methods

### Collection of hosts

Our main field site for the collection of ladybirds was the 480-ha land of the USDA Southeastern Fruit and Tree Nut Research Laboratory in Georgia, USA^66^, where we collected specimens of *Harmonia axyridis* and *Olla v-nigrum* in 2014–2015. In addition, ladybirds were collected by the first author or collaborators at different sites in four continents: Africa, Asia, Europe and North America (details in Supplementary Table S2). Sampling of ladybirds was done using a variety of standard entomological methods: Tedders pyramidal traps, light traps, hand collecting and sweeping in stands of weedy vegetation along the banks of swamps and small lakes and at the sides of roadways. Long-time preservation of ladybird specimens was in 95% ethanol at −20 °C. In addition to field-collected material, pinned ladybirds in dried insect collections were screened for the presence of Laboulbeniales. The Coccinellidae collection of the Boston Harbor Islands All Taxa Biodiversity Inventory project at the Harvard Museum of Comparative Zoology (Cambridge, MA), the Division of Invertebrate Zoology at American Museum of Natural History (New York City, NY) and the Florida State Collection of Arthropods (Gainesville, FL) were primary sources for infected ladybirds.

### Collection of Laboulbeniales

Preserved insects were examined for the presence of Laboulbeniales under a dissecting microscope at 10–50× magnification. *Hesperomyces* thalli were removed from their hosts using Minuten Pins (BioQuip, #1208SA, Rancho Dominguez, CA) inserted onto wooden rods. Following standard procedure by Benjamin^67^, we removed thalli or groups of thalli and mounted them in Amann’s medium, a liquid solution. Before applying Amann’s medium and to facilitate microscopic observations, thalli first had to be arranged and fixed onto the microscope slide. To make thalli a bit sticky, they were first placed in a droplet of Hoyer’s medium (30 g arabic gum, 200 g chloral hydrate, 16 mL glycerol, 50 mL ddH_2_0). Next, thalli were individually picked up and arranged in one or two rows. After a brief period of drying, the slide was closed using a cover slip with a drop of Amann’s medium (drop facing downward) and subsequently sealed with nail polish or B-72 in acetone (Gaylord, #AB72, Syracus, NY). We viewed mounted specimens at 400–1000× magnification using an Olympus BX40 microscope equipped with an XC50 camera (Olympus, Waltham, MA). Identification was done using descriptions from Thaxter^64,68^, Santamaria^69^ and De Kesel^63^. Slides are deposited at Farlow Herbarium (FH; Harvard University, Cambridge, MA).

### Morphological studies

To assess morphological variation in thalli we took measurements of 22 parameters per thallus (Fig. 1): total length of the thallus including haustorium (total L w foot, point a—point x in Fig. 1), total length of the thallus (total L, b—x), length of cell I (L cell I, b—d), width of cell I (W cell I, c—o), length of cell II (L cell II, m—o), width of cell II (W cell II, l—n), length of cell III (L cell III, d—f), width of cell III (W cell III, e—l), total length of receptacle (total L rec., b—f), length of basal cell of the appendage (L bas. app., f—g), total length of appendage (total L app., f—k), length of longest antheridium (L lngst. anth., h—j), length of longest antheridial neck (L anth. neck, i—j), length of cell VI (L cell VI, m—z), width of cell VI (W cell VI, p—y), perithecium length (L perith., w—z), perithecium width (W perith., r—x), length of second tier of perithecial wall cells (tier II, q—r), third tier (tier III, r—s), fourth tier (tier IV, s—t), length of lobes (lobes, t—w) and length of longest projection (lngst. proj., u—v). To correct for natural variation in length and width, these ratios were calculated: L/W cell I, L/W cell II, L/W cell III, total L rec./total L, total L app./total L, L/W cell VI, tier II/L perith., tier III/L perith., tier IV/L perith., lobes/L perith., L/W perith., L perith./total L and lngst. proj./L perith.

Measurements were made at 400–1000× magnification with cellSens Standard 1.14 software (Olympus) using the Polyline measuring tool. We measured at least 30 adult thalli from each host population. Maturity was judged by the presence of ascospores within the perithecium. To exclude potential position-induced morphological variation^62^, only thalli from the elytra were measured and used in this study.

We analyzed variation in morphology of thalli from different host species and populations using generalized linear mixed models (GLMM), implemented in the R package *lme4*^70^. Random effects for insect specimen were included, because we measured several thalli from the same host individuals. Hypothesis testing was done using likelihood ratio tests, with *P*-values calculated based on chi-squared distributions, declaring an effect significant when *P* ≤ 0.05. Two models were compared for each variable, the null model (Mod0) and the model with host species as explaining variable (Mod1). Model selection happened using the Akaike Information Criterion^71^. For a selection of variables with significant differences between host species in the GLMMs, principal component analysis (PCA) followed by exploratory biplots were made. PCA was only done for ratios to visualize variation in shape and structure independent of size. PCA and biplots were obtained using the R package *factoextra*^72^.

### DNA extraction methods

We extracted DNA from 1–18 *Hesperomyces* thalli either using the QIAamp DNA Micro Kit (Qiagen, Stanford, CA), a modified Extract-N-Amp Plant PCR kit (Sigma-Aldrich) procedure^73^ or a modified REPLI-g Single Cell Kit (Qiagen, St. Louis, MO) protocol^74^. The QIAamp DNA Micro Kit protocol was followed as per the manufacturer’s instructions. One major change we implemented was the increase of the incubation time at 56 °C for complete lysis, to several days. With the Extract-N-Amp Plant PCR kit, thalli were removed at the foot with a tiny drop of Hoyer’s medium or glycerin at the tip of a Minuten Pin and placed in a 0.5 µL PCR tube filled with 20 µL of Extraction Solution. The tube was incubated at room temperature for 10–30 min and then at 95 °C for 20 min. The extract was then diluted with 60 µL of Dilution Solution (3% Bovine Serum Albumin). The REPLI-g Single Cell Kit is different from the previous protocols because it adds a whole-genome amplification (WGA) step to the DNA isolation, thus providing a considerable benefit when material is scarce. A Minuten Pin was submerged in glycerin to remove a single thallus from its host and place it in a droplet of glycerin on a microscope slide. The thallus was carefully placed in a 0.2 mL PCR tube with 2 µL of phosphate-buffered saline (PBS). These steps were done at 40× magnification under a stereomicroscope. After adding 1.5 µL of prepared D2 buffer, the tube was incubated at 65 °C for 20 min. Subsequent steps followed the manufacturer’s instructions. All steps of this procedure were performed under a laminar flow hood to ensure sterile conditions.

For a majority of our isolates, we applied pre-treatments to increase the likelihood of successful isolation and subsequent PCR amplification. These pre-treatments included subsequent cycles of freezing on liquid nitrogen and heating to 95 °C, prolonged incubation at 56 °C in 180 µL ATL buffer + 20 µL proteinase K or in 20 µL Extraction Solution using a Shake ‘N Bake Hybridization Oven (Boekel Scientific, model no. 136400–2, Feasterville, PA) and homogenization in a FastPrep FP120 Cell Disrupter at 5.0 m/sec for 15 s (Thermo Fisher Scientific, Waltham, MA). For both the QIAamp DNA Micro Kit and the Extract-N-Amp Plant PCR kit, we often manually crushed thalli in 1.5 mL tubes using a 1.5 mL pellet pestle (Kimble, #749521-1500, Vineland, NJ). In the REPLI-g Single Cell Kit, the single thallus was often cut in half through the perithecium using a sterile no. 10 surgical blade on a disposable Bard-Parker handle (Aspen Surgical, Caledonia, MI) before placing it in the 0.2 mL PCR tube.

### PCR amplification and DNA sequencing

We amplified the nuclear small and large ribosomal subunits (SSU and LSU) and the internal transcribed spacer region of the ribosomal DNA (ITS). Primer combinations used were NS1/NS2, NS1/NS4, SL122/SR4, NSL1/NSL2, SL122/NSL2 and SL344/NS6 for SSU^73,75,76^; ITS1f/ITS4, ITS1f/ITS4A and ITShespL/ITShespR for ITS^74,75,77,78^; and LIC24R/LR3 and LR0R/LR5 for LSU^48,49^. PCR reactions consisted of 2.5 µL of each 10 µM primer, 13.3 µL of RedExtract Taq polymerase (Sigma-Aldrich), 5.7 µL of ddH_2_O and 1 µL of DNA extract. For all amplifications, an Eppendorf Mastercycler ep gradient thermocycler was used with initial denaturation at 94 °C for 3:00 min; followed by 35 cycles of denaturing at 94 °C for 1:00 min, annealing at 50 °C for 0:45 min, extension at 72 °C for 1:30 min; and a final extension step of 72 °C for 10:00 min. When PCR reactions were unsuccessful, we optimized PCR conditions to include multiple annealing temperatures^79^: initial denaturation at 95 °C for 10 min; followed by 30 cycles at 95 °C for 1 min, 62 °C for 1 min (decreasing 1 °C every 3 cycles) and 72 °C for 1:30 min; then 30 cycles at 95 °C for 30 s, 55 °C for 30 s and 72 °C for 1 min; and a final extension step of 72 °C for 7 min. PCR products that showed clear bands on agarose gel were purified using the QIAquick PCR Purification Kit (Qiagen) and subsequently sequenced. We prepared 10 μL reactions with the same primers and 3–5.5 μL of purified PCR product. The sequencing reactions were performed using the Big Dye® Terminator v3.1 Cycle Sequencing Kit (Life Technologies, Carlsbad, CA). Generated sequences were assembled, trimmed and edited in Sequencher 4.10.1 (Gene Codes Corporation, Ann Arbor, MI).

### Sequence alignment and phylogenetic analyses

We constructed 3 datasets, ITS, LSU and a concatenated SSU+ITS+LSU dataset, to investigate phylogenetic structure within *H. virescens*. We aligned sequences of each region separately using Muscle v3.7^80^, implemented on the Cipres Science Gateway^81^. For SSU and LSU, ambiguously aligned regions and uninformative positions were detected and removed using trimAl v1.3^82^ with 60% gap threshold and minimal coverage of 50%. In the ITS dataset, we manually removed the ITS1 (positions 1–525) and ITS2 (687–1067) spacer regions for those sequences other than *Hesperomyces* because they were too variable to align. The data for each region were concatenated in MEGA7^83^ to create a matrix of 2767 bp with phylogenetic data for 52 isolates.

Maximum likelihood (ML) analyses were run using PAUP on XSEDE^84^ in Cipres^81^. Nucleotide substitution models were selected statistically with the help of jModelTest^85^ by considering the Akaike Information Criterion (AIC). For the ITS dataset, the TVM+G model was selected (lowest -lnL = 3566.8229). For the LSU dataset, the TIM1+G model gave the best scoring tree (-lnL = 3151.3620). ML was inferred for each dataset under the appropriate model; rapid bootstrap (BS) analysis was implemented with 100 replicates.

We performed ML and Bayesian analyses for the SSU+ITS+LSU dataset. For ML, the dataset was divided into three partitions. The best substitution model was selected using jModelTest^85^ by considering the Akaike Information Criterion (AIC). The following models were selected: TPM2uf (SSU), TVM+G (ITS) and GTR+G (LSU). [We did not use all LSU isolates for the three-gene concatenated dataset, hence the selection of a different substitution model.] ML was inferred under partitioned models using IQ-tree^86,87^; ultrafast bootstrap (BS) analysis was implemented with 1000 replicates^88^. Also for Bayesian inference, the three-gene dataset was divided into partitions. Analyses were done with a Markov chain Monte Carlo (MCMC) coalescent approach implemented in BEAST^89^, with an uncorrelated lognormal relaxed molecular clock allowing for rate variation across the tree. We selected the Birth-Death Incomplete Sampling speciation model^90^ as tree prior with JC (for SSU), TPM2uf+G (for ITS) and TIM1+G (for LSU) nucleotide substitution models (considering the Bayesian Information Criterion from jModelTest) and a lognormal ucld.mean (mean = 5.0, stdev = 1.0). Five independent runs were performed from a random starting tree for 10 million generations, with a sampling frequency of 1000. Prior settings were entered in BEAUti^89^ to generate an XML file, which was run using the BEAST on XSEDE tool in Cipres^81^ (three runs) and locally from the command line (two runs). The resulting log files of the five runs were entered in Tracer v1.6.0^91^ to check trace plots for convergence and effective sample size (ESS). Burn-in was adjusted to achieve ESS values of ≥200 for the majority of sampled parameters. While removing a portion of each run as burn-in, log files and trees files were combined in LogCombiner. TreeAnnotator was used to generate consensus trees with 0% burn-in and to infer the maximum clade credibility tree, with the highest product of individual clade posterior probabilities. Final trees with bootstrap values (BS) and posterior probabilities (pp) were visualized in FigTree v1.4.3 (http://tree.bio.ed.ac.uk/software/figtree/).

### Species delimitation analyses

Morphology-based identification of *H. virescens* thalli^63,64,68,69^ may mask multiple species within a geographical context or with strict host specificity. Therefore, we used 3 species delimitation methods to validate species hypotheses^28,92,93^: Automatic Barcode Gap Discovery (ABGD) and General Mixed Yule Coalescent methods (GMYC), and a Poisson tree processes (PTP) model approach.

ABGD is based on the detection of a “barcode gap,” which is observed when nucleotide divergence among isolates of the same species is smaller than divergence among isolates of different species in a given multiple alignment. Gaps are identified and used to partition (or: split) the data into the maximum number of groups, which represent species hypotheses^92^. We used the web version of ABGD (at http://wwwabi.snv.jussieu.fr/public/abgd/abgdweb.html) to identify barcode gaps in the SSU+ITS+LSU dataset. Genetic distances were calculated using both available distance metrics (JC69, K80) with the following parameters: Pmin = 0.001, Pmax = 0.01, steps = 10 and Nb bins = 20. To assess consistency of the species recognized by ABGD, we evaluated results for four gap width values (X): 0.1, 0.5, 1.0 and 1.5.

In the PTP model approach, the number of nucleotide substitutions is directly used to model speciation rate. The underlying assumption is that the number of substitutions between species is significantly higher than the number of substitutions within species^28^. Compared with GMYC, Zhang and colleagues^28^ found that PTP performs best especially when the evolutionary distances between species are small. PTP is intended for the delimitation of species in single-gene trees. As a result, we applied this method to both the ITS and LSU phylogenetic reconstructions separately. As input for the PTP model approach, we used phylogenetic trees generated by Bayesian analyses for the two datasets. The MCMC analyses were done under a strict molecular clock, with the Yule speciation tree prior and the appropriate nucleotide substitution model, as selected by the Bayesian Information Criterion from jModelTest 2.1. For the ITS dataset, the TVM2uf+G model was selected (lowest -lnL = 3573.5434). For the LSU dataset, the K80+I model gave the best scoring tree (-lnL = 1672.9035). Two independent runs were performed from a random starting tree for 10 million generations, with a sampling frequency of 1000. The two resulting log files were combined in LogCombiner with 1% burn-in. Consensus trees with 0% burn-in were generated and the maximum clade credibility tree was constructed in TreeAnnotator. We used the bPTP web server (http://species.h-its.org). The “b” in bPTP stands for the Bayesian support values that are added to delimited species. The different parameters were set as default (number of MCMC generations, thinning, burn-in, seed). For both analyses the outgroups were removed from the dataset prior to constructing the phylogenetic tree.

GMYC uses a fully-resolved ultrametric tree^93^ inferred from a single marker to model processes at the population level (coalescence) and processes at the species level (speciation). As input we used the ITS and LSU maximum clade credibility trees generated for PTP. In addition, we reconstructed a maximum clade credibility tree in BEAST^89^ using the concatenated SSU+ITS+LSU dataset. For this analysis we removed the outgroups from the dataset, because the inclusion of distantly related species makes it more difficult for GMYC to detect closely related species. The MCMC analysis was done under an uncorrelated lognormal relaxed molecular clock, with the Birth-Death Incomplete Sampling speciation model^89^ tree prior, the GTR+I+G nucleotide substitution model (as selected under the Bayesian Information Criterion) and a lognormal ucld.mean (mean = 5.0, stdev = 1.0). Two independent runs were performed from a random starting tree for 80 million generations, with a sampling frequency of 8000. The two resulting log files were combined in LogCombiner with 10% burn-in. The maximum clade credibility tree was again constructed in TreeAnnotator. Species were delimited based on this generated ultrametric tree with the GMYC method using R packages *rncl*^94^ and *splits*^95^.

## Electronic supplementary material

Supplementary Table S1

Supplementary Table S2

## Data Availability

Voucher specimens of infected ladybirds will be deposited at the Harvard Museum of Comparative Zoology, Cambridge, MA and the Brabant Museum of Nature in Tilburg, The Netherlands. Voucher slides of *Hesperomyces virescens* are deposited at Farlow Herbarium at Harvard University (FH) (barcodes available in Supplementary Table S1). All generated sequences have been uploaded to GenBank (accession numbers MG745336–MG745358, MG757798–MG757831, MG760581–760611). The following data are available from figshare 10.6084/m9.figshare.c.3944749.v1: sequence alignments generated during this study (in NEXUS format), input XML and output log and trees files from Bayesian analyses, R code used for the GMYC analysis.
