# Supplementary material for: Integrative taxonomy reveals hidden species within a common fungal parasite of ladybirds
Source: Sci Rep. 2018 Oct 29;8:15966. doi: 10.1038/s41598-018-34319-5 (PMC6206035; doi:10.1038/s41598-018-34319-5)
Supplement: Supplementary file 2 — Supplementary Table S2 [file 41598_2018_34319_MOESM2_ESM.pdf]

Integrative taxonomy reveals hidden species within a common fungal parasite of ladybirds

Danny Haelewaters, André De Kesel, Donald H. Pfister

Supplementary Table S1. Overview of host specimens used during this study, with locality information and names of *H. virescens* isolates taken from these hosts. Geographic coordinates in parentheses are not exact. *Laboratory colony of *O. v-nigrum* originated from pecan orchards in Georgia. The colony was housed at the OEB Greenhouse, Harvard University.

Host species	Country	Locality	Geographic coordinates	Isolate(s)
Adalia bipunctata	Denmark	Region Hovedstaden, Copenhagen, Kongens Have	55°41'06.8" 12°34'48.1"	D. Haelew. 1193a
Adalia bipunctata	Italy	Umbria Region, Perugia	(43°06'38.52" 12°23'26.88")	D. Haelew. 1231a, 1232a, 1247a
Adalia bipunctata	Sweden	Stockholm, Strandvägen and Humlegården	(59°20'08.7" 18°04'57.4")	D. Haelew. 1199h
Adalia decempunctata	Italy	Umbria Region, Perugia	(43°06'38.52" 12°23'26.88")	D. Haelew. 1248b, 1249a
Azya orbigera	Panama	Chiriquí Province, Potrerillos Abajo	8º38'55.48" -82º28'14.10"	D. Haelew. 928g

Cheilomenes propinqua	South Africa	Western Cape Province, Stellenbosch	-33º56'18.96" 18º52'20.53"	D. Haelew. 653a, 655c, 659b, 659d, 1259a
Cycloneda sanguinea	Panama	Chiriquí Province, Boquete, Jaramillo Abajo	8º44’46.84” -82º25’34”	D. Haelew. 924a
Cycloneda sanguinea	Panama	Chiriquí Province, Volcán, Urbanización La Florida	8º47’23.47” -82º38’44.93”	D. Haelew. 929a
Halyzia sedecimguttata	Netherlands	North Brabant Province, Tilburg, nature reserve De Kaaistoep	51°32' 5°01'	D. Haelew. 955b
Harmonia axyridis	Germany	Bavaria Free State, Gereuth	50º7'33.55" 10º49'5.92"	D. Haelew. 646a, 646c
Harmonia axyridis	Japan	Honshu, Kansai Region, Kyoto	34°59'09.9" 135°45'31.5"	D. Haelew. 1268b, 1268d
Harmonia axyridis	Netherlands	Utrecht Province, IJsselstein, Randdijk	52º1'34.24" 5º3'43.06"	D. Haelew. 334b
Harmonia axyridis	Netherlands	North Brabant Province, Tilburg	51º33'22" 5º4'7"	D. Haelew. 361a
Harmonia axyridis	Netherlands	North Brabant Province, Tilburg, Sportpark Merlinello	51º34’46.2” 5º4’11.3”	D. Haelew. 1174a

Harmonia axyridis	South Africa	Western Cape Province, Stellenbosch	-33º55'58.86" 18º51'36.55"	D. Haelew. 648c, 669a, 943a, 943b, 1005c
Harmonia axyridis	USA	Georgia, Peach County, Byron, USDA Southeastern Fruit and Tree Nut Research Laboratory	32º39'28.31" -83º44'15.05”	D. Haelew. 316a/d, HM497c
Harmonia axyridis	USA	Massachusetts, Boston Harbor Islands National Park Area, Plymouth County, World's End peninsula	42º15'51.9" -70º52'37.8"	D. Haelew. 486c
Harmonia axyridis	USA	Massachusetts, Bristol County, North Easton, Greenspade Inn	42°03'55.8" -71°06'32.2"	HM516a
Harmonia axyridis	USA	Massachusetts, Middlesex County, Cambridge, William James Hall	42°22'38.0964" -71°06'48.5316"	D. Haelew. 1188g
Olla v-nigrum	USA	Georgia, Peach County, Byron, USDA Southeastern Fruit and Tree Nut Research Laboratory	32º39'28.31" -83º44'15.05”	JP352b, 353a, 353b, 354b; D. Haelew. 954d*, 954e*, 1200h*, 1200i*
Psyllobora vigintimaculata	USA	California, Alameda County, Oakland, Peralta Park	37°47'50.478" -122°15'37.08"	D. Haelew. 1250b, 1250c, 1251b

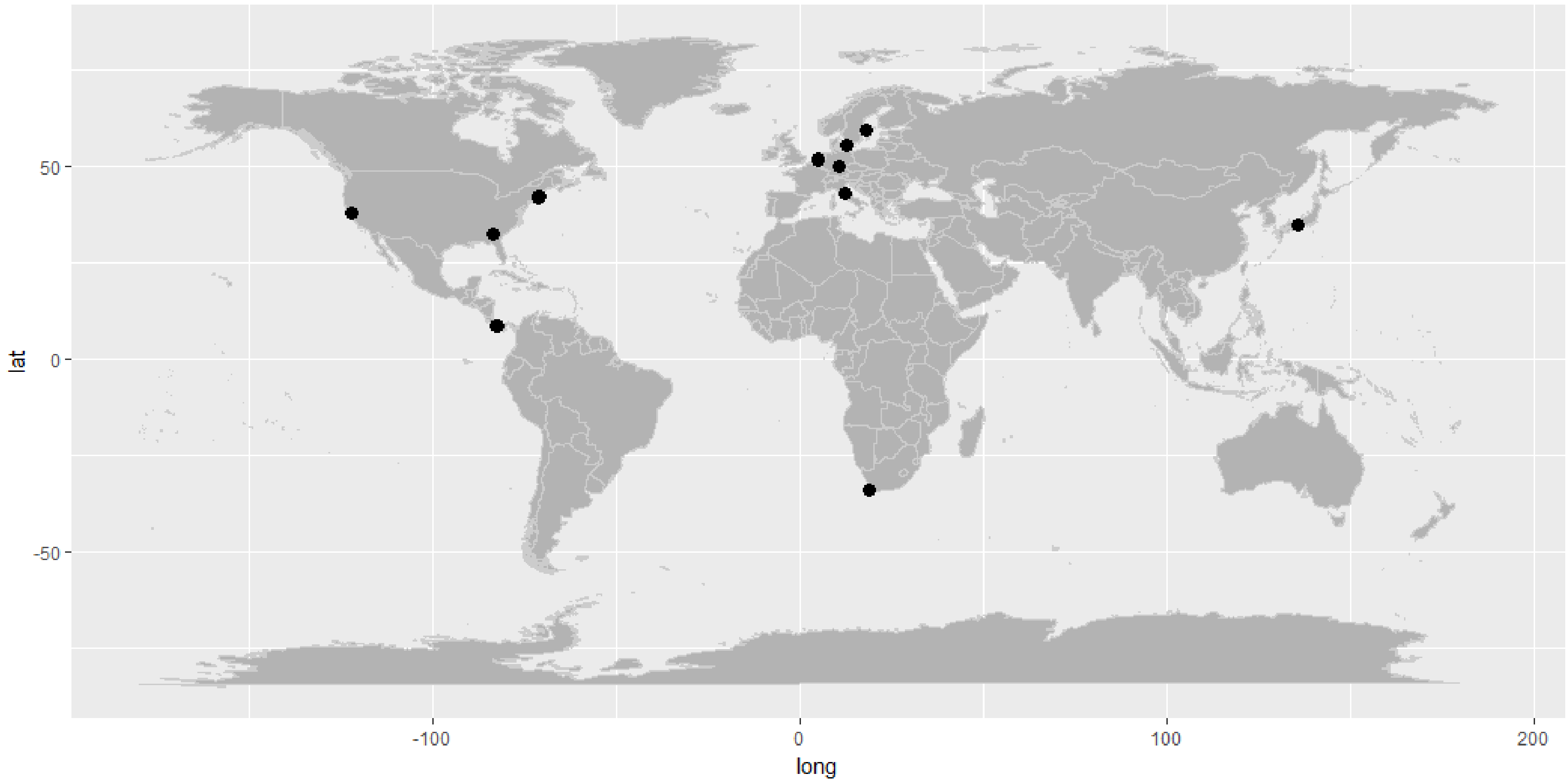

50
0
-50
lat
-100
0
100
200
long
